# Energy-Aware QoS MAC Protocol Based on Prioritized-Data and Multi-Hop Routing for Wireless Sensor Networks

**DOI:** 10.3390/s22072598

**Published:** 2022-03-29

**Authors:** Aan Nazmus Sakib, Micheal Drieberg, Sohail Sarang, Azrina Abd Aziz, Nguyen Thi Thu Hang, Goran M. Stojanović

**Affiliations:** 1 Department of Electrical & Electronic Engineering, Universiti Teknologi PETRONAS, Seri Iskandar 32610, Perak, Malaysia; mdrieberg@utp.edu.my (M.D.); azrina_aaziz@utp.edu.my (A.A.A.); 2 Faculty of Technical Sciences, University of Novi Sad, Trg Dositeja Obradovića 6, 21000 Novi Sad, Serbia; sohail@uns.ac.rs (S.S.); sgoran@uns.ac.rs (G.M.S.); 3 Department of Telecommunications Networks, Faculty of Telecommunications, Posts and Telecommunications Institute of Technology (PTIT), Hanoi 100000, Vietnam; hangntt@ptit.edu.vn

**Keywords:** WSNs, quality of service, energy-aware, multi-hop routing

## Abstract

Wireless sensor networks (WSNs) have received considerable interest in recent years. These sensor nodes can gather information from the surrounding environment and transmit it to a designated location. Each sensor node in WSN typically has a battery with a limited capacity. Due to their large number and because of various environmental challenges, it is sometimes hard to replace this finite battery. As a result, energy-efficient communication is seen as a critical aspect in extending the lifespan of a sensor node. On the other hand, some applications that require large coverage and generate various sorts of data packets require multi-hop routing and quality of service (QoS) features. Therefore, in order to avoid network failure, these applications need an energy-efficient QoS MAC protocol that can support multiple levels of data packet priority and multi-hop routing features while focusing on energy conservation. An energy-aware QoS MAC protocol based on Prioritized Data and Multi-hop routing (EQPD-MAC) is proposed in this article. The EQPD-MAC protocol offers a simple yet effective cross-layer communication method. It provides timely delivery of multi-priority packets, uses an adaptive active time to limit idle listening, and integrates a robust routing protocol. Finally, the EQPD-MAC protocol’s performance was evaluated and compared to three other well-known QoS MAC protocols. The simulation findings show that the proposed protocol significantly decreases sensor node energy consumption by up to 30.3%, per-bit energy consumption by up to 29.6%, sink node energy consumption by up to 27.4% and increases throughput by up to 23.3%.

## 1. Introduction

Wireless sensor networks (WSNs) are composed of tiny, low-powered sensors capable of detecting many types of urgent information from their surroundings, and have the primary features of robustness, scalability, and large-scale deployment capability [1,2]. These miniature devices can sense and extract information from the environment and are often referred to as sensor nodes. These nodes are capable of storing physical data, performing computational tasks, and forwarding the gathered information to a specific point [3,4]. They are used for a wide range of applications, including healthcare, residential, smart homes, industrial, military, and other commercial purposes [5,6,7]. Sensor nodes in WSN have finite battery life and it is challenging to replace and recharge the battery in a timely manner [8]. This becomes prohibitively expensive when a large number of nodes are spread out across a vast area [9].

When it comes to ensuring effective communications in WSNs, Medium Access Control (MAC) protocol plays an important role. When the MAC protocol is well designed, it allows the sensor nodes to access the medium to improve energy efficiency while minimizing latency [10]. The Quality of Service (QoS) plays a vital role and refers to the capability to assign alternative priority to data packets depending on the requirements of the user [11]. End-to-end latency, energy efficiency, network throughput, and packet delivery ratio are essential QoS characteristics. Sensor nodes should provide data packets with varying levels of urgency or priority, for example, fire alerts have a higher priority than temperature monitoring [12,13].

Routing’s function is to find the best way to get data from one place to another. The network layer is extensively utilized in WSNs to handle the routing of incoming data, and the routing protocol is a significant aspect of the architecture of a communication stack [14]. In multi-hop networks, intermediate sensor nodes are responsible for relaying packets to the base station. Therefore, there must be several paths for data to go from source to the destination that guarantees reliability. Furthermore, due to the limited battery life, energy conservation is a critical design consideration in WSNs [15].

Researchers have done extensive work in the past to save energy, focusing primarily on optimizing MAC protocols [16], routing protocols [17], data acquisition [18], and cross-layer optimization approaches [19]. However, most of the energy is used at the MAC layer in sensing the channel, idle listening, packet transmission, packet reception as well as overhearing, and data collisions [20,21]. It is also important to note that the routing protocols in WSNs are application-specific and energy-efficient [22]. A good routing protocol must be simple, energy-aware, adaptable, and scalable [23,24].

A typical deployment of many sensor nodes has brought various design and management issues for sensor networks. Because of the unbalanced traffic, the sensor node’s energy is depleted far sooner than it should be. The primary design purpose of WSNs is to facilitate data transmission while attempting to extend the network’s life [25]. In WSNs, an essential performance parameter is the network lifetime, which is defined as the amount of time until the energy in the first sensor node runs out [26].

Considerable enhancements have been made to many QoS-aware MAC protocols [27] to improve energy efficiency, network lifetime, support packets’ priority in the network and so on. Some of the prominent MAC protocols that consider QoS characteristics are EAMP-AIDC [28], AQSen-MAC [29], MPQ-MAC [30], QAEE-MAC [31], PMME-MAC [32], RMP-MAC [33] and our previously proposed the TMPQ-MAC protocol [34]. However, all these protocols lack the multi-hop routing feature, which limits the deployment area to the transmission range. On the other hand, MAC protocols with multi-hop routing have also been proposed, such as MDA-SMAC [35], SPEECH-MAC [36], EDS-MAC [37], and DCD-MAC [38]. Nevertheless, the protocols mentioned above have limited consideration in supporting the QoS features and have not prioritized different types of sensor data packets, thus limiting their use to only applications with homogeneous data.

Therefore, this paper proposes an Energy-Aware QoS MAC protocol for Multi-hop and Prioritized Data in WSNs (EQPD-MAC), that provides both QoS and multi-hop routing features. Additionally, the receiver employs a mechanism that reduces latency for the highest priority data packets and enhances the network’s energy efficiency. The performance of the EQPD-MAC is compared with MPQ-MAC [30], PMME-MAC [32], and RMP-MAC [33].

The following is a list of the contributions made by this work:

- The EQPD-MAC takes into account data packets with multiple priorities.
- AODV routing is incorporated to enable multi-hop routing and integrated with the MAC to ensure an energy-efficient protocol.
- A novel approach that provides best energy efficiency while still maintaining good end-to-end latency.
- In order to assess the performance of EQPD-MAC, real-world measurements are used.
- EQPD-MAC provides improved energy consumption, average end-to-end latency, throughput, and packet delivery ratios.

The remainder of the paper is organized as follows: The discussion and summary of related works on WSN from Application and MAC layer standpoints are provided in Section 2. In Section 3, the description of the proposed QoS MAC protocol with multi-hop routing feature, EQPD-MAC, is given. Details of the design and implementation at the Application, Network, and MAC layer levels are also provided. Section 4 presents the extensive performance evaluation, discussion and analysis of the EQPD-MAC and other comparative protocols. Finally, Section 5 concludes the work.

## 2. Related Works

The Internet of Things (IoT) is continuously emerging and is gaining interest in many applications, including smart cities, structural health, and industrial systems. Many (WSN) applications in the IoT context create heterogeneous data that require differentiated service, and these applications need ways to combine three functions into one: sensing, processing, and communicating. The connection between sensor nodes is established by a series of choices made at several communication levels, such as assigning priority at the application layer, energy awareness mechanism at the MAC layer and the routing protocol at the network layer. Usually, the priority is assigned by the user’s applications but the authors in [39] proposed an interest forwarding strategy where the priority of the packet is assigned by following a probabilistic approach using Content-Centric Networking.

### 2.1. Application Layer

The application layer provides the user with the interfaces they need in order to engage with the real world through the WSNs. The application layer is also responsible for transforming data into a readable format for the purpose of detecting actual information. Assigning a priority at the application layer guarantees that all nodes are aware of the priority when delivering the packet, allowing for quicker transmission of the critical events.

When developing a new protocol, prioritization is an effective strategy for improving network performance. Identifying events of interest in sensor networks is a very effective method of exploiting priorities in sensor networks. A strategy to prioritize sensor-based applications in smart cities is presented in [40], which can be differentiated based on their monitoring goals, certain contextual prioritization characteristics and the detection of critical events. Data packets generated by sensor nodes comprise critical and non-critical events across the range of applications. Critical events must be sent to the closest emergency responders with the shortest possible delay and must be prioritized above non-critical or less urgent events [41]. FROG-MAC [42] introduced a new fragmentation strategy for heterogeneous traffic in WSNs that enables high-priority data packets to interrupt any unimportant ongoing transmissions.

QoS refers to the ability to prioritize data packets based on the user’s needs. QoS prioritizes emergency situations such as fire threats, volcanic eruptions, medical emergencies, leaks, and hazardous gas detection [43,44,45]. In contrast to less urgent events, higher priority ones often need better QoS, which means less delays and higher packet success rates. The authors in [46] proposed a QoS-aware cross-layer configuration that aims to establish a network configuration which satisfies application-specific process requirements while also adhering to user-defined QoS restrictions on event-transport reliability. Because of the wide range of possible uses for WSNs, the system requirements may differ significantly from one application to another. Table 1 summarizes the QoS needs for various applications.

### 2.2. MAC Layer

Energy efficiency may be accomplished at a number of different layers, from application layer to routing layer and finally to the MAC layer. In WSNs, the MAC layer, which serves as the wireless channel acquisition technique, has the highest impact on the amount of energy consumed [47,48]. The MAC protocol is in charge of controlling the radio module functionalities, i.e., transmit, receive, and listen, to coordinate sensor node access to the shared wireless medium [49]. Due to the limited resources, capacities of sensor nodes, and the features of wireless communication, achieving a good trade-off between all of these is very difficult. Since the radio module is the most energy-intensive component of a sensor node, the MAC protocol specifies a sleep schedule that allows the radio modules of sensor nodes to be placed in sleep mode as much as possible throughout the day [50,51]. As a result, an energy-efficient MAC protocol must be developed that satisfies the real-time needs of WSNs.

#### 2.2.1. QoS MAC Protocols in WSNs

For the purpose of providing QoS, a variety of MAC protocols have been proposed for WSN, such as QAEE-MAC [31], MPQ-MAC [30], PMME-MAC [32], RMP-MAC [33], EAMP-AIDC [28], etc.

High-priority packets are sent first using the QoS Aware Energy-Efficient MAC (QAEE-MAC) protocol [31]. The protocol only takes into account two levels of data packet priority. The protocol’s analytical evaluation assumes just a single node that sends high-priority packets, whereas the rest of the network’s nodes produce low-priority packets, which may not be practical. Aside from that, QAEE-MAC does not provide multi-hop data packet transmission or other energy-saving strategies.

Multi-priority QoS MAC (MPQ-MAC) [30] allows multiple levels of data packet priority. Because of this, considerable changes to the protocol were introduced to increase energy efficiency while maintaining packet priority in the network. The sink node keeps track of the waiting timer depending on the importance of the incoming packets in this method. Receiving several Tx-beacons (transmitter beacons) during the waiting period allows the receiver node to prioritize senders. While MPQ-MAC takes QoS into account, it does not provide multi-hop communication for data packets.

Multi-event Priority MAC Protocol (PMME-MAC) [32] allows multiple priorities by assigning corresponding probability values for channel accessibility. High-priority nodes can expect reliable and low-latency from PMME-MAC, based on its mathematical analysis. PMME-MAC solves the issue of concurrent priority transmission of critical packets. However, the cost of the low latency comes in terms of high power consumption, and it also lacks multi-hop transmission.

Receiver-Initiated and Multi Priority Backoff MAC (RMP-MAC) [33] protocol proposed adaptive backoff methods depending on the event priority, remaining energy level and the amount of data packets waiting to be sent. Energy usage is reduced because the transmitter adjusts its schedule depending on information from the receiver’s wake-up time. Emergency data packets are delivered more quickly by taking the advantage of the adaptive backoff mechanism. Even though RMP-MAC considers the QoS characteristics, it does not allow multi-hop routing feature for data packets.

Energy-Aware MAC Protocol with Adaptive Individual Duty Cycle (EAMP-AIDC) [28] prioritizes data packets that need to be delivered quickly. The protocol’s goal is to minimize energy waste via dynamic individual duty cycle. It attempts to maximize the active time of each node based on their energy budget, distance from the sink, and network congestion. The protocol uses a routing protocol, which allows each potential relay (PR) nodes to have a selectors list. The list contains nodes (cluster members) whose data packets can be relayed by the PR. Even though EAMP-AIDC considers multi-hop communication, data packets can only be relayed if the sensor nodes are on the PR list. Sensor nodes that are not on the list have to wait until other nodes complete their packet transmissions. The protocol only considers two packet priorities. Only cluster members generate high priority packets that must be transmitted directly to the sink, increasing energy consumption.

#### 2.2.2. MAC Protocols with Multi-Hop Routing Feature in WSNs

Numerous researchers have spent considerable time developing novel routing methods to address multiple issues in WSNs [52]. The on-demand distance vector routing protocol (AODV) [53,54] is a reactive routing protocol and is one of the most commonly utilized in the industry in which each node acts as a router. AODV obtains and maintains routes only when they are required [55]. The protocol’s fundamental activities include route discovery, establishment of forward and reverse paths, routing table management, control of local connections, and path maintenance.

In order to conserve energy, the Special Purpose Energy-Efficient Contention-based Hybrid MAC (SPEECH-MAC) [36] protocol incorporates the AODV routing protocol and the dual-hop approach. The sink node uses the dual-hop approach to collect data directly from the child nodes for any urgent packet and then collects data from the relay nodes where other child nodes can forward their data packets to the relay nodes. However, the protocol does not provide any energy-saving strategies for the child nodes that are going to use the dual-hop approach and might die rather soon because of their distance from the sink.

Division-Multiple Access-MAC (MDA-SMAC) [35] is another protocol that is based on the AODV routing protocol. MDA-SMAC is an extension to the S-MAC protocol that incorporates two backoff techniques. The Fast-Binary Exponential Backoff and Conflict-Avoid-Binary Exponential Backoff algorithms are used to minimize data latency, which further lowers the chance of conflict. The duty cycle is constantly adjusted by the nodes based on the length of the buffer queue. MDA-SMAC achieves lower latency, higher throughput, and better residual energy than the S-MAC protocol by the combined efforts of backoff algorithms and micro-duty. Furthermore, the protocol utilizes the AODV routing protocol for multi-hop communication but does not take into account the QoS features of data packets.

In EDS-MAC [37], two phases are provided in the proposed method: data transfer and cluster formation. In the first step, the VSSFFA algorithm generates energy-aware clusters by selecting the best cluster heads. Several factors go into the selection process: residual energy, intra-cluster distance, the number of potential cluster heads, and node degree. The data transmission stage is used for data transfer, which decreases latency, and control overhead. The protocol only considers two levels of data packet priority. The protocols analytical evaluation assumes that sender nodes which require minimum latency or minimum duty cycle to reach sink node will have the highest priority, which is not very realistic.

Comparative analysis is shown in Table 2 for all the protocols mentioned above. On one hand, some QoS protocols lack multi-hop routing features, while the other protocols that provide multi-hop routing lack QoS features for data packets. Although a small number of protocols support both QoS and multi-hop routing, they lack higher packet priority for different kinds of emergencies. In addition, their performance assessments have not taken into account all performance metrics, such as energy efficiency, end-to-end packet latency, network throughput, or packet delivery ratio. As a result, there is a need to develop an energy-aware QoS MAC protocol that considers multi-hop communication for WSNs. It should have the ability to send data packets with multiple priority levels and can be deployed to cover a large area, with good energy efficiency.

## 3. EQPD-MAC Protocol Development

### 3.1. Method

This section provides the operation of the EQPD-MAC protocol when several sensors attempt to deliver data packets to the sink and multiple rounds of the data packet transmission. This section also describes Activation Time (TA) and the communication process of the highest priority packet transmission.

#### 3.1.1. Communication of EQPD-MAC

The EQPD-MAC protocol uses *p*-persistent Carrier Sense Multiple Access (CSMA) technique and a synchronized approach for the data packet transmission, with QoS and multi-hop routing features. It uses an adaptive active/sleep time to limit idle listening in order to reduce the energy consumption. It is capable of maintaining an optimal activation time (TA), which is set dynamically under a wide range of loading situations. The proposed EQPD-MAC protocol has the ability to extend or terminate the TA whenever needed. If the sensor nodes are idle for a specific time, the nodes simply go to sleep in order to conserve energy. It can also handle up to four levels of packet priority, although the number of levels can be extended to any value.

**Figure 1:**
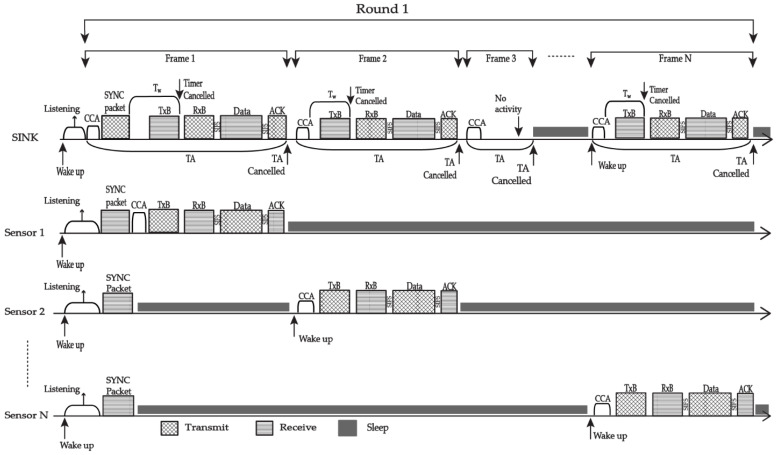
The communication process of EQPD-MAC.

The EQPD-MAC protocol’s communication process is shown in Figure 1. The sensor nodes start listening to the channel as soon as they wake up. After a certain amount of time, all sensor nodes broadcast a SYNC packet that provides the start schedule time of the next frame. The sensor nodes will adopt both schedules if the sink delivers another SYNC packet that differs from the sensor node schedule. As soon as the synchronization with the sensor nodes is complete, TA will be initiated by the sink. It will wait for the Tx-beacon from the sensor node at its scheduled time for the transmission of the data packet until the waiting timer (*T_w_*) ends. If the sink receives a sudden Tx-beacon with P_4_ priority from a different sensor, the sink node will cancel the waiting timer and broadcast an Rx-beacon with the address of the selected sensor. The sink will wait for the intended packet after transmitting the Rx-beacons and will go to sleep if it does not receive a Tx-beacon at the end of *T_w_*.

**Figure 2:**
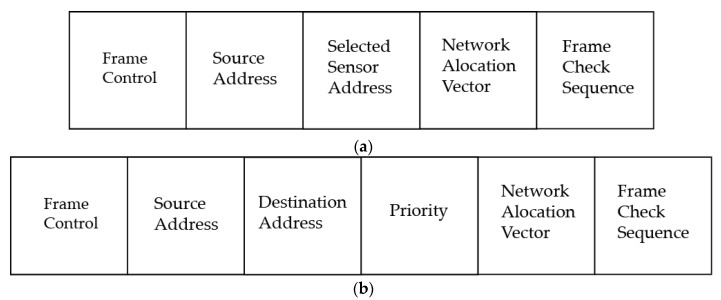
Packet format of Rx-beacon and Tx-beacon (**a**) Rx-beacon format. (**b**) Tx-beacon format.

Meanwhile, Tx-beacon will be sent to the sink node once the sensor node has completed Clear Channel Assessment (CCA) and the medium is idle for the transmission. After that, the node will expect an Rx-beacon from the sink. When the sensor node receives Rx-beacon, it examines the beacon and checks whether it contains the sensor’s address. The priority packet will be transmitted if Rx-beacon includes the address, and if it does not, the sensor will go directly to sleep to save energy. After dispatching the priority packet, the sensor will wait for an acknowledgement packet (ACK) as a receipt for the successful transmission of the packet, goes to sleep, and cancels the TA. The TA can be renewed automatically if the data packet transmission is not completed, and the node receives either a Tx-beacon or an Rx-beacon, whose packet formats are shown in Figure 2.

**Figure 3:**
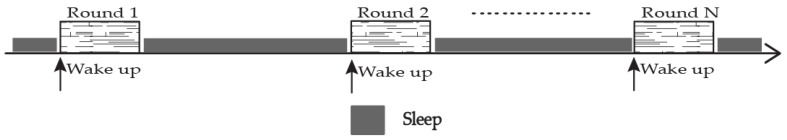
Multiple rounds of data transmission.

Referring to Figure 1, the operation of EQPD-MAC when several sensors attempt to deliver data packets to the sink, is described as follows. Each of the sensor nodes have adopted the sink’s schedule after the synchronization. Sensor 1 will send its packet in frame 1, Sensor 2 will send its packet in frame 2 and so on. Sensor 1 will execute CCA and transmit Tx-beacon for the data packet transmission. After that, Sensor 1 will expect Rx-beacon from the sink, and upon receiving it, the node will inspect the beacon to see if it has been chosen to send the data packet. Since it is chosen, Sensor 1 will send its data packet and wait for an ACK packet before returning to sleep. TA will be automatically terminated after a successful data packet transmission. After that, Sensor 2 and other sensor nodes will wake up at their predetermined time, execute CCA, and transmit Tx-beacon for the data packet transmission. TA will also be automatically terminated if there is inactivity during the TA period. After completing each frame time, the sink goes to sleep and concludes the first round. The sink sleeps after each round of data packet transmission, as shown in Figure 3.

#### 3.1.2. Activation Time (TA)

The sensor node and the sink node automatically adopt one another’s schedules. At the start of each frame, the sensor nodes will begin their *TA*. Sometimes it is possible that the data packet transmission remains unfinished during a *TA*. When a node gets a Tx-beacon or an Rx-beacon, the *TA* may be renewed to finish the data packet transmission. In order to receive an Rx-beacon packet, the *TA* should be long enough. The *TA* has the following lower limit [56].

(1) $$TA\;>\;C+\;T_{PKT}+T$$

where *C* is the duration of the contention interval, $T_{PKT}\;$ is the length of a Tx-beacon, and *T* is the time between the end of the Tx-beacon packet and the beginning of the Rx-beacon packet. The nodes may use this strategy to prolong and terminate the active duration, to avoid energy waste due to idle listening. The *TA* expires automatically, and the sink node goes to sleep in case of no activity to reduce idle listening and energy consumption.

#### 3.1.3. Highest Priority Packet Transmission

The proposed EQPD-MAC protocol supports multi-priority transmission and employs a technique to minimize the latency of the highest priority packet. The EQPD-MAC has four distinct priority levels for data packets, as shown in Table 3: normal, important, very important, urgent. This is extensible to any number of priority levels.

**Figure 4:**
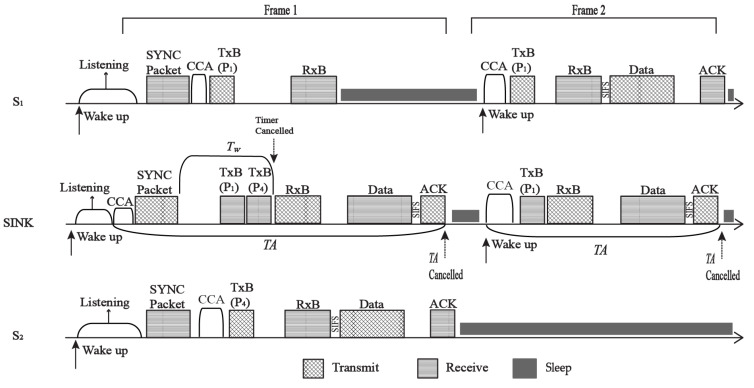
Priority packet transmission.

Consider a case of two sensor nodes, S_1_ and S_2_, that try to send their respective priority data packets to the sink, as shown in Figure 4. In order to send the packets, S_1_ and S_2_ must first synchronize with the sink. After that, S_1_ and S_2_ perform CCA and transmit Tx-beacon with P_1_ and P_4_, respectively. At the sink, the timer is immediately cancelled since S_2_ has the highest priority packet. The sink sends an Rx-beacon containing the source address of S_2_. After receiving the Rx-beacon, S_1_ and S_2_ will inspect the beacon. Since Rx-beacon contains S_2_ as the selected sender, P_4_ packet will be sent to the sink node at frame 1. In the meantime, S_1_ will be forced to go to sleep and is given another scheduled time at frame 2 to send its P_1_ packet. After receiving the priority packet from S_2_, the sink node sends an ACK packet as reception of a successful transmission. This method of transmitting the P_4_ priority packet instantly minimizes latency and conserves energy by allowing other sensor nodes to sleep.

### 3.2. Algorithm

**Figure 5:**
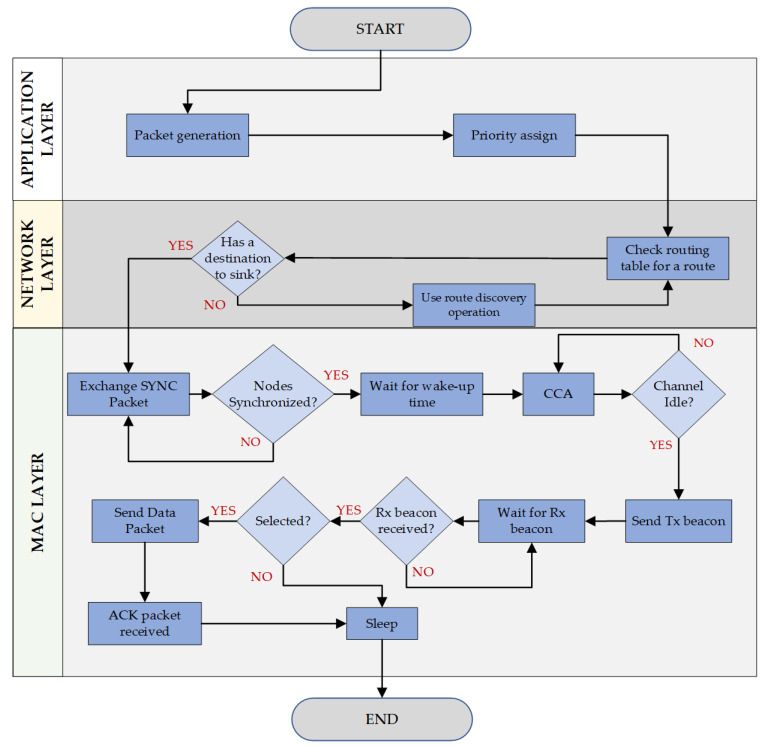
Design of the Application, Network, and MAC layer levels.

The EQPD-MAC protocol design and development involves the whole protocol stack in each layer to facilitate communication. The three-layer protocol consisting of Application, Network, and MAC layers, is as represented in Figure 5.

Priority of packets are implemented at the application layer of the network stack, as priority is determined by the particular application, e.g., emergency alert signal. In EQPD-MAC, an extra priority field have been added to the application, which ensures that all nodes are aware of the priority while transmitting the packet. The actual end-to-end latency of the packet can be accurately measured by assigning priority to application layer. Data fields such as source ID, destination ID, and sequence number are defined on the application layer. Subsequently, the application layer communicates with the network layer to transmit priority data packets. With the help of the network layer, packets can be routed to the sink node by establishing a topology of the network so that each node knows its own location and its neighbors. Following that, the packet is passed to the MAC layer by the network layer.

The MAC layer is especially crucial to the proposed protocol since it manages the packet in accessing the channel and incorporates the technique of adaptive active/sleep time to lower the energy usage. It interacts with the network layer to transmit and receive data packets and also to identify whether the overheard packets are meant for the node. The communication process of the MAC layer is already shown in Figure 1. The nodes that could not synchronize with the sink node will go to sleep and wake up in the following round to detect the sink node’s schedule by anticipating the reception of the SYNC packet. The sensor nodes will continue this process until it is able to synchronize with the sink node. When the MAC layer is in broadcast mode, it is important to obtain additional information from the upper layers so that a node may determine if each received packet is meant for itself or not. Decapsulating every received packet and transferring it to the network layer, the EQPD-MAC protocol employs a simple but effective cross-layer communication. If a packet has been overheard, the network layer reports to the MAC protocol that the packet has been discarded. Upon receipt of an ACK packet from the destination node, the MAC layer forwards this information to the network layer, which subsequently forwards it back to the application layer.

#### Multi-Hop Routing Communication

The sensor nodes that are far away from the sink will select other sensor nodes to relay the data packet to the sink. The EQPD-MAC protocol uses the widely known AODV routing protocol to relay the packets. The routing protocol’s essential activities include path discovery, routing table management, forward and reverse path configuration, path maintenance, and local connectivity management. At the beginning, AODV launches a route discovery operation for all the sensor nodes. AODV maintains the next hop routing information in a route table.

**Figure 6:**
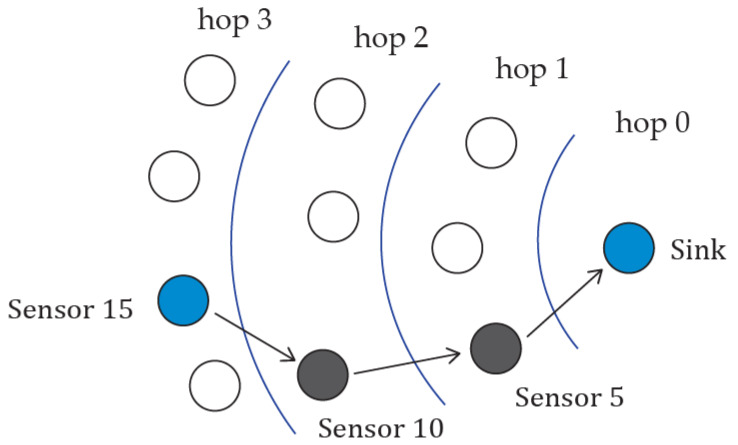
Multi-hop Topology.

Figure 6 shows the multi-hop topology of the proposed EQPD-MAC protocol. After each sensor node knows a route to the sink node, nodes will wake up and send their respective priority data packets to the sink using the path stored in the route table. For example, Sensor 15 will wake up and perform CCA to see if the medium is idle. The sensor node needs other sensor nodes which can relay the data packet. Sensor 15 checks the routing table to see if it has a path to the sink node. If it does not have a path, it launches the route discovery operation, and finds that the sink is three hops away. In this case, Sensors 10 and 5 are the intermediate sensor nodes to relay the data packet to the sink.

If the medium is idle, Sensor 15 transmits Tx-beacon and T_w_ is stopped. After receiving the Tx-beacon, Sensor 10 will broadcast an Rx-beacon with the sensor’s address indicating its availability to receive and relay the data packet. Otherwise, it will simply go to sleep and wait for the next round of data packet transmission.

When Sensor 15 receives Rx-beacon, it examines the beacon and checks whether it contains the sensor’s address. After that, the priority data packet will be transmitted by Sensor 15. Next, the sensor will wait for an ACK as a receipt for the successful transmission of the packet and goes to sleep. The ACK packet is overheard by Sensor 5, and it will directly receive the priority packet from Sensor 10 after performing CCA. Sensor 5 will route the priority packet to the sink and after receiving the intended priority packet the sink sends an ACK packet.

### 3.3. Energy Model

The energy model is described in this subsection. The energy expended when a node switches from receiving to transmitting mode or vice versa, is called the switching energy. Hence it is given by:

(2) $$E_{Switching}{\;=P}_{Switching}\;\times \;T_{Switching}$$

where $P_{Switching}$ and $T_{Switching}$ denote the power consumption and the amount of time it takes a node to do the switching, respectively.

In order to ensure that the channel is clear of other transmissions, each node inspects it before transmitting. Listening energy refers to the energy used to examine the channel. In listening mode, the radio is operational but does not receive or transmit any packets.

(3) $$E_{Listening}{=P}_{Listening}\;\times \;T_{Listening}$$

where $P_{Listening}$ and $T_{Listening}$ denote the power consumption and the amount of time it spent listening to the channel, respectively.

When a node sends a Tx beacon or receives an Rx beacon, the amount of energy consumed is given by:

(4) $$E_{Beacon}=P_{Beacon}\;\times \;T_{Beacon}$$

where $P_{Beacon}$ and $T_{Beacon}$ denote the power consumption and the amount of time it takes to send or receive a beacon, respectively.

Transmission energy is dependent on the distance travelled and the amount of interference encountered. Packets are exchanged between the nodes in this situation, either by transmitting or forwarding them by the other nodes. Total energy consumed during each packet transmission is given by:

(5) $$E_{TX}=\;L_{Pkt\;}{\times \;P}_{TX}{\;\times \;T}_{TX}$$

where $L_{Pkt}$, $P_{TX}$ and $T_{TX}$ denote length of each packet in bytes, the power consumption, and the amount of time it takes a node to do a transmission, respectively.

When a node receives packets, it expends energy in the same way as when it transmits.

(6) $$E_{RX}=\;L_{Pkt\;}{\times \;P}_{RX}{\;\times \;T}_{RX}$$

where $L_{Pkt}$, $P_{RX}$ and $T_{RX}$ denote length of each packet in bytes, the power consumption, and the amount of time it takes to receive a packet, respectively.

Furthermore, the amount of energy wasted by the nodes when they switch off after the node goes to sleep state is also given by:

(7) $$E_{Sleep}{\;=P}_{Sleep}\;\times \;T_{Sleep}$$

where $P_{Sleep}$ and $T_{Sleep}$ denote the power consumption and the amount of time it takes to go into the sleep state, respectively.

As a result, the overall energy consumption $E_{T}$ can be calculated by adding up the energy used and is given by:

(8) $$E_{T}=\overset{n}{\underset{i=0}{\sum }}P_{i}{\times T}_{i}$$

Assuming that there are *n* states, *i* is the number of the particular state in which the radio is on, and *P* is its power consumption rate, *T* is the amount of time spent in each state *i* etc.

The equations show that the time spent in each state affects energy usage. As the time spent in each stage increases, the energy consumption will increase. In other words, if the sensor nodes can process packets more quickly, the energy consumption can be reduced. Additionally, the nodes save energy during idle states by extending the sleeping duration through adaptive active/sleep time. EQPD-MAC dynamically changes the amount of time spent listening and sleeping in order to reduce energy usage.

### 3.4. Castalia Implementation and Simulation

**Figure 7:**
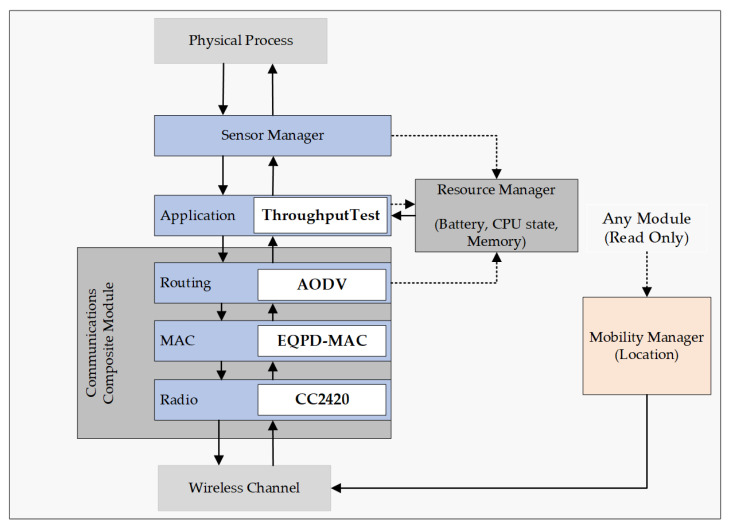
The node composite module.

Both the TMAC [56] and the MPQ-MAC [30] protocols inspired the development of the EQPD-MAC. Castalia Simulator [57] provides the implementation of the TMAC protocol inside the MAC module directory. Every feature of EQPD-MAC protocol were implemented by modifying the TMAC protocol, which supports adaptive active/sleep time. TMAC protocol supports the activation timeout (*TA*). The internal construction of the node composite module is shown in Figure 7. Message passing is represented by solid arrows, whereas dashed arrows represent the basic function call.

1. Priority is implemented inside “ThroughputTest” to support multi-priority data packets. “ThroughputTest” is an application module inside the Castalia directory. Each packet is assigned a random value between 0 and 1, representing its priority. P_4_ is the most important, whereas P_1_ is the least important priority packet.
2. Data fields such as source ID, destination ID, and sequence number are defined in the application layer. The actual end-to-end latency of the packet can be accurately measured by assigning priority in the application layer.
3. After that, to access the channel, another random number is generated inside the MAC module. If the random number is greater than p, then the sensor node can begin the data packet transmission.
4. The sink node first starts the waiting timer T_w_ to receive the Tx-beacons. The waiting timer had to be implemented inside the MAC module.
5. The sensor nodes send the Tx-beacons, and the beacon contains the priority number. The sink node checks the priority and selects a sensor node based on the higher priority. Such conditions of checking the priority have to be implemented inside the code.
6. The sink node sends an Rx-beacon containing the address of the selected sensor node, and the timer is canceled. The sensor node receives Rx-beacon and inspects if the beacon contains its address. If the source address matches the sensor address, the node sends the priority packet and waits for an ACK packet from the sink.
7. In Castalia, the TMAC module provides several basic functions such as startup, toNetworkLayer, timerFiredCallback. These functions should also include the priority field, ensuring that all nodes are aware of the priority while transmitting the packet.
8. AODV routing module also provides several basic functions such as startup, fromApplicationLayer, fromMacLayer, timerFiredCallback, toMacLayer. The priority field has to be implemented on all functions, ensuring that all nodes are aware of the priority while transmitting the packet.
9. After that, AODV routing protocol was tested with the EQPD-MAC protocol and the performance was evaluated. The transmissions of the packets can be viewed as an output using the “trace” command inside the code. Additionally, the AODV routing protocol code is available [58].

#### 3.4.1. Implementation Challenges

To implement priority in the proposed MAC protocol, several functions had to be modified in Application, MAC, and Network layer, which is the most difficult part because in Castalia, it is necessary for a new protocol to derive from predefined base/virtual classes. These virtual classes have already established the methods and structure to implement new protocols that a developer may and should utilize. These Virtual classes are namely: VirtualApplication, VirtualMAC and VirtualRouting. So, priority had to be defined inside each of the virtual classes.

EQPD-MAC had to be tested with some of the routing protocols available in Castalia in order to support the multi-hop features of the data packets so that the proposed MAC protocol could achieve the maximum energy efficiency possible. To incorporate the multi-hop feature in the proposed MAC protocol, several functions in the AODV had to be modified as well. This is because AODV only keeps track of the source ID and destination ID. When the neighboring node receives the data packets, it checks the source and destination. After the implementation of the priority into AODV, the neighboring nodes can also check the priority field. In this way the higher priority data packets can be sent much quicker.

#### 3.4.2. Simulation Process

The simulation runs for 10,000 s in a 200-by-200-m area and comprises 46 nodes, 45 of which are randomly distributed. The SINK node, which has the ordinal number of 0, is located at the center of the sensor field.

1. First, navigate into the directory Castalia/Simulations/radioTest, where the omnetpp.ini file is located. Modules such as Routing, Application, and MAC protocol must all be included in the simulation’s omnetpp.ini file. The file allows the simulation to be executed in Castalia. The simulation parameters are recorded in this file. For example, the simulation area, the total number of sensor nodes, the simulation period, the packet rate, the initial energy, and so on.
  sim-time-limit = 10,000 s
  SN.field_x = 200
  SN.field_y = 200
  SN.numNodes = 46
  SN.deployment = “[0]->center;[1..45]->uniform”
2. In order to start the simulation, open a terminal window in Ubuntu and change the directory to the ~/Castalia/Simulations/radioTest$ and enter the following commands:
  ../../bin/Castalia -c EQPD, AODV
3. Castalia creates two files once the simulation is complete: a text file (e.g., 220117-030436.txt) and a CastaliaTrace file. The text file provides information on the overall energy consumption of each sensor node, the number of packets received, the initial energy, and so on. CastaliaTrace file contains all the events of interest that was specified in the code to be viewed, mainly for verification and validation purposes. The EQPD.cc file is the main source of the program’s code from which trace instructions can be included. CastaliaTrace, for example, may contain whether the Tx-beacon or Data packet is transmitted or, if the Rx-beacon is received from the sink node, or to see the computation of the average end-to-end latency of the packets, etc.
4. To observe the packet delivery ratio and how much energy each node consumed, the following instructions are typed into the terminal window in Ubuntu.
  ../../bin/CastaliaResults -i 220117-030436.txt -s energy
  ../../bin/CastaliaResults -i 220117-030436.txt -s packets

After each simulation run, the results are verified in this manner.

#### 3.4.3. Validation

Packets are first generated at the Application layer, which also assigns their priority. When the packet arrives at VirtualApplication, it sets the source and destination. The application then uses toNetworkLayer command to shift the packet into VirtualRouting, which sends the packet to AODV routing protocol. AODV routing protocol receives the packet at fromApplicationLayer function and checks the priority with other information, e.g., source ID, destination ID, and encapsulates the packet with all the information. The routing protocol then checks the routing table if it has a destination. If it does not have any route to the destination, it will use route discovery operation and finds the route. AODV uses the command toMacLayer to pass the packet to the MAC layer after finding the destination.

The packet is received by the MAC layer using the fromNetworkLayer function. When packets arrive to the MAC layer, they are decapsulated and checked for priority, source ID, and destination ID by VirtualMAC. A Tx-beacon is then sent by the MAC either to the sink node or the next hop in the network. If the Tx-beacon is received by the sink node, then according to the synchronization time, the sink will wake up and receives the priority packet by exchanging the Rx-beacon, data packet and followed by ACK packet. After the successful transmission of the data packet, the MAC will pass a short frame using toNetworkLayer command that packet has been received by sink and Network layer receives this frame at fromMacLayer. The Network layer decapsulates the frame to verify the source ID and destination ID. After the verification, the frame is passed to the source node that originated the packet to inform that the packet has been received by the sink node. This frame is eventually being received by the Application layer using the command fromNetworkLayer and total end-to-end latency is measured after the frame arrives at the Application layer.

If the next hop is an intermediate node, then the priority packet is passed to the Network Layer using the command toNetworkLayer. Data packets are sent straight to the next hop by exchanging Tx-beacon and Rx-beacon using toMacLayer command. The hop count will be increased by one for each time a packet is received by an intermediary node. All the packets pass, and results can be validated in the CastaliaTrace file by using the command “trace”.

The findings are validated with other protocols after each successful execution of the code. Considering the case when 45 sensor nodes are scattered randomly around the sensor field, EQPD-MAC consumes 0.2945 mJ/bit whereas PMME-MAC, MPQ-MAC and RMP-MAC consume 0.3819, 0.3519, and 0.3619 mJ/bit energy per sensor node, respectively. The average end-to-end latency of EQPD-MAC is 0.1274 s whereas the latency for PMME-MAC, MPQ-MAC and RMP-MAC is 0.1650, 0.3730, and 0.3439 s, respectively. The PDR is almost 100% for both EQPD-MAC and PMME-MAC whereas the PDR for MPQ-MAC and RMP-MAC is 85% and 76%, respectively. Throughput is around 10,000 bps for both EQPD-MAC and PMME-MAC whereas throughput is only 8600 bps and 7649 bps for MPQ-MAC and RMP-MAC, respectively. These results are validated against the published results of the comparative works.

## 4. Results and Discussion

Castalia 3.3 [57] and OMNET++ 4.6 [59] simulators were used to evaluate the performances of EQPD-MAC and comparative works. The simulation adopts parameters from TelosB [60] and MicaZ [61] sensor node and CC2420 radio, which is a commonly used low-power RF transceiver in WSN hardware motes. Receiving and idle operation both incur 62 mW of power, whereas transmitting and sleeping operation use 46.4 mW and 1.4 mW of power, respectively. To provide reliable wireless connectivity, the radio chipset uses the 2.4 GHz frequency spectrum to transmit data at 250 kbps over a distance of around 50 m.

The sink is located at the center of the sensor field of 200 m × 200 m square region, with 45 nodes scattered around randomly. Packets with length of 28 bytes are generated by each sensor node. In Castalia, the data are completely random. Each packet is assigned a random value *(R)* between 0 and 1, which represent its priority. Each time, a random number between 0 and 1 is generated and it is used to differentiate between priority packets, as shown in Table 4. Each priority has a 25% probability of occurring in the simulation, which means that all priorities will have equal possibilities. In order to acquire a clear understanding of the network’s performance and to ensure that all nodes get the same amount of all priority packets, such circumstances were used to conduct the simulation. However, we have also tested the proposed protocol with P_4_ having 5%, P_3_ having 15%, P_2_ having 30% and P_1_ having 50% probabilities, which may represent a different application. The outcomes are almost identical.

TelosB and MicaZ uses TinyOS as their platform. TinyOS has a default data field size of 28 bytes. In WSNs, there are packets of varying sizes and types, and the priority of these packets differ from one another. Smaller packets are preferred to larger ones because the retransmission of a long packet that is lost will be much more energy extensive. Packet header, data, and trailer are the three primary elements of most network packet formats. In order to transmit packets, the physical layer uses the packet header and trailer (MAC layer information). To ensure a reliable transmission, the MAC layer might split a large data file into smaller ones before sending it. Small packets, on the other hand, can be transmitted quickly and reliably. As a result, a reduction in total energy consumption is achievable.

Before the sensor node transmits Tx-beacon, it creates a random value, and if the produced value is less than *p*, it transmits. The AODV routing protocol is used to route data packets from sensor nodes to a sink node in this network, through multiple hops. Table 5 contains the simulation parameters. The simulation runs for 10,000 s which ensures that at least 10,000 packets are simulated. The simulator was used to assess the proposed protocol’s performance and comparison with three other widely used MAC protocols. All the protocols use *p*-persistent CSMA to transmit Tx-beacons and data packets.

### 4.1. Energy Consumption Per-Bit

The average energy consumption per-bit, *E*, may be calculated by dividing the total energy consumption by the multiple of the total number of packets received and the length of each packet. *E* is defined by the following Equation (9):

(9) $$E=\frac{E_{T}}{{NP}_{PktRx}{\;\times \;L}_{Pkt}}$$

In this Equation, $E_{T}$, ${NP}_{PktRx}$ and $L_{Pkt}$ represent the total energy used, the overall packets received by the sink, and length of each packet, respectively. 

**Figure 8:**
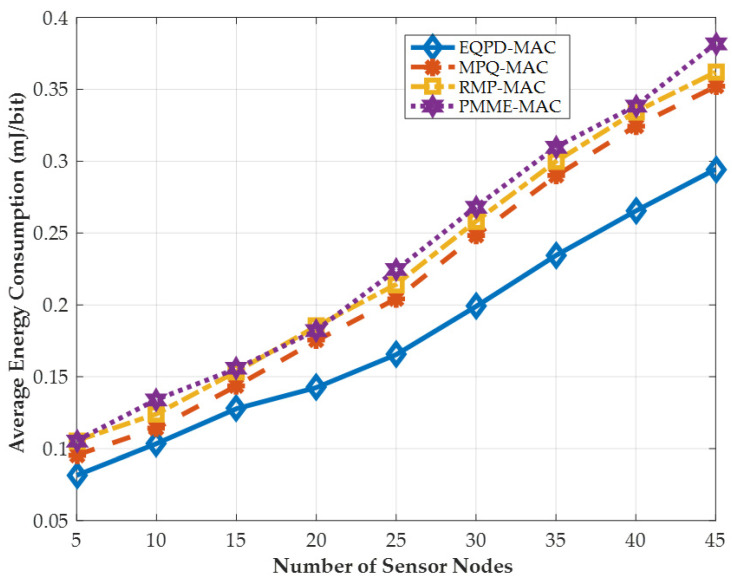
Average energy consumption per-bit.

Figure 8 shows the average energy consumption per-bit, and the performance of the proposed EQPD-MAC protocol is compared with PMME-MAC, MPQ-MAC, and RMP-MAC. The figure shows that all protocols consume less energy while there are fewer sensor nodes, but as the number of nodes increases, the energy usage increases since the sensor nodes must deal with more packets. It can be seen that the proposed EQPD-MAC gives the best performance, especially when number of sensor nodes increases. When there are 5 sensor nodes, EQPD-MAC consumes 0.0815 mJ/bit whereas PMME-MAC consumes 0.1056 mJ/bit energy per sensor node. When there are 45 sensor nodes, EQPD-MAC consumes 0.2945 mJ/bit whereas PMME-MAC consumes 0.3819 mJ/bit energy per sensor node. Compared to PMME-MAC, MPQ-MAC, and RMP-MAC, the EQPD-MAC provides improvements by up to 29.6%, 24.8%, and 29.5%, respectively. Firstly, the reason is because EQPD-MAC decreases the power consumption at all sensor nodes by using adaptive active/sleep time. Secondly, after receiving the SYNC packet, sensor nodes synchronize with other sensor nodes including the sink and extend their sleep time. On the other hand, PMME-MAC has been seen to consume the most energy among all the protocols. It is because PMME-MAC lacks the ability for the sensor nodes to conserve energy by going to sleep automatically while not participating and prioritizes packet latency above energy conservation.

### 4.2. Energy Consumption per Sensor Node

**Figure 9:**
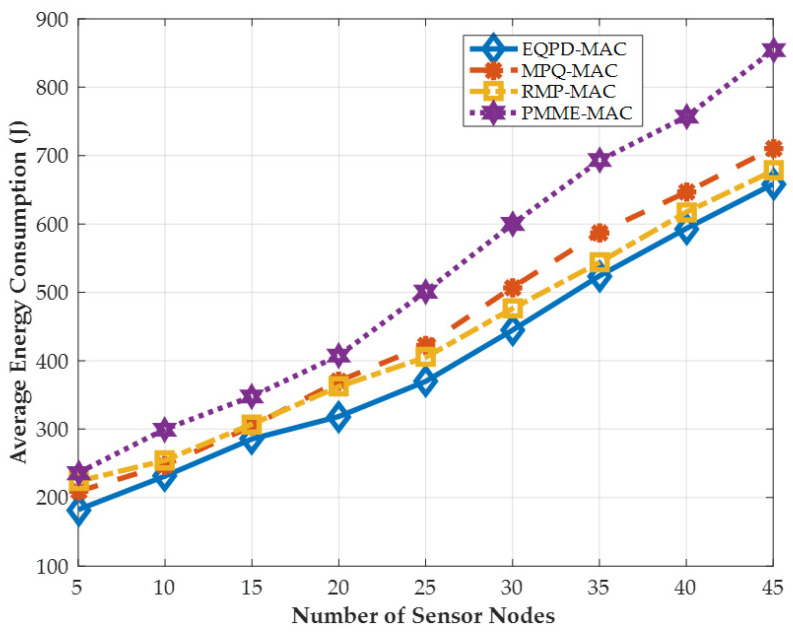
Average energy consumption per sensor node in Joule.

Figure 9 shows the average energy consumption. The trend is similar to the previous figure, and it can be noted that EQPD-MAC outperforms MPQ-MAC, RMP-MAC, and PMME-MAC by up to 7.5%, 7% and 30.3%, respectively. However, it may seem that percentage gains are not as high especially against MPQ-MAC and RMP-MAC. This is because EQPD-MAC transmits more packets than these protocols, which consumes more energy. Nevertheless, EQPD-MAC still provides the best performance, and it is able to provide this while delivering the highest number of packets.

### 4.3. Sink Energy Consumption

**Figure 10:**
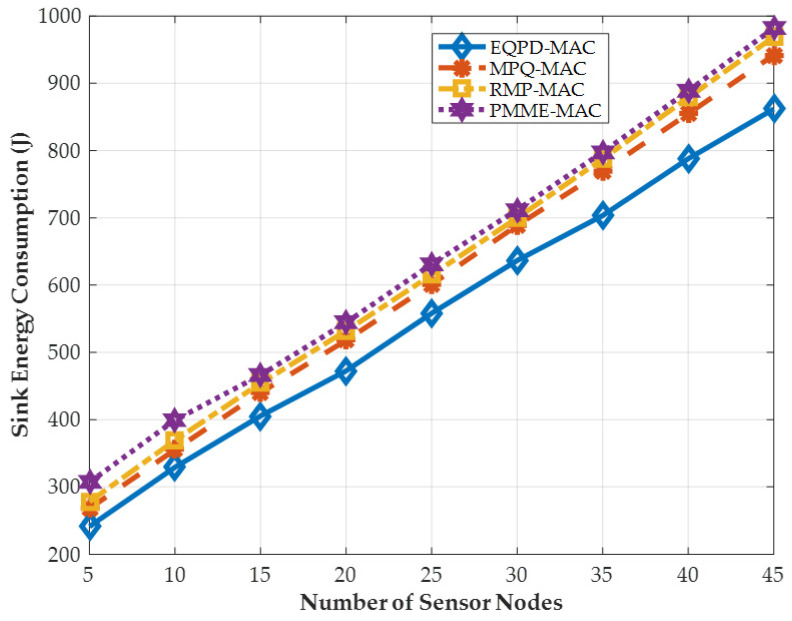
Sink energy consumption in Joule.

Figure 10 shows the sink/base station energy consumption. In comparison to other protocols, it can be seen that the EQPD-MAC delivers a considerable decrease in energy consumption of up to 27.4%. The sink node uses adaptive active/sleep time with the help of TA, which helps preserve more energy. RMP-MAC, on the other hand, automatically changes the duty cycle based on the energy level of the nodes and includes an adaptive backoff mechanism to maximize energy savings in the network. When there are fewer sensor nodes, the protocol reduces the energy consumption. However, as the number of sensor nodes increases, it begins to lose packets, resulting in increased energy consumption. This is because when the sender nodes’ buffer limit is surpassed, the incoming data packets are dropped. In MPQ-MAC and PMME-MAC, the sink node does not provide any method of conserving energy and the sink nodes rarely sleep, causing its remaining energy to decline rapidly.

### 4.4. End-to-End Latency for Priority Packets

In communication network, the end-to-end latency of a packet is measured as the time between its generation and reception at its destination. The formula for calculating the average end-to-end latency is shown below.

(10) $$d_{ETE\;}{=d}_{queu}{+d}_{trans}{+d}_{proc}{+d}_{prop}$$

There are four different latencies in this equation: the latencies associated with queuing, transmitting, processing, and propagation. In this equation, $d_{queu}$, $d_{trans}$, $d_{proc}$, and $d_{prop}$ signify the queuing time, the transmission time, the processing time, and the propagation time, respectively.

**Figure 11:**
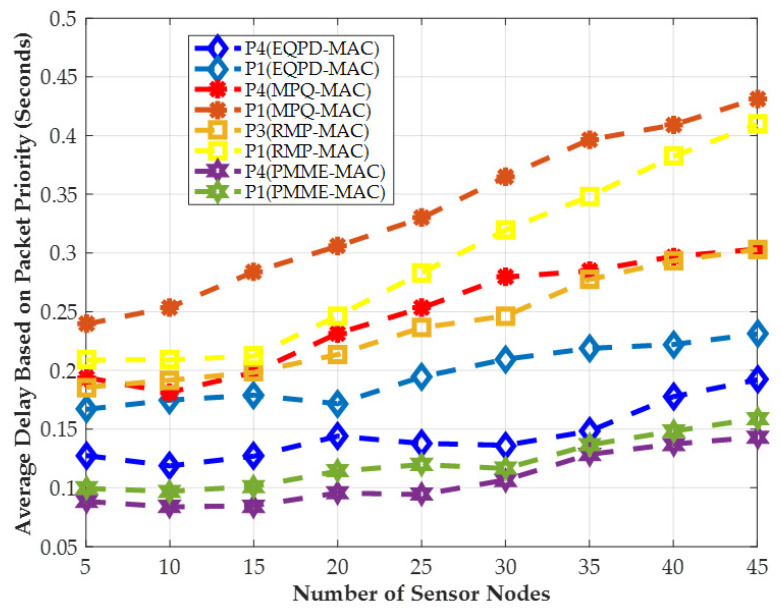
Average end-to-end latency of priority packets.

Figure 11 shows the average end-to-end latencies of EQPD-MAC, MPQ-MAC, PMME-MAC, and RMP-MAC for the highest and lowers priority data packets. According to the results, EQPD-MAC has a 75% reduction in latency for P_4_ priority packets compared to MPQ-MAC. EQPD-MAC protocol also transmits P_4_ priority packets more quickly than RMP-MAC’s highest priority level (P_3_) by up to 74%. Compared to the other protocols, MPQ-MAC has the highest latency because the nodes must wait longer for the waiting timer *T_w_* to expire, resulting in the higher latency. It can be seen that there is a noticeable difference in the priority packet latency between the PMME-MAC and EQPD-MAC protocols. PMME-MAC does not consider any technique to minimize the energy consumption and achieves reduced end-to-end latency since the sensor nodes never sleep, which increases the energy consumption of the sensor nodes. However, to prevent any network disruption, the EQPD-MAC uses the TA mechanism to conserve more energy at the cost of a marginal increased latency. Despite this, the EQPD-MAC can still support the highest priority packet to provide an acceptable latency for priority packets, while maintaining the lowest energy consumption.

### 4.5. Average End-To-End Latency

**Figure 12:**
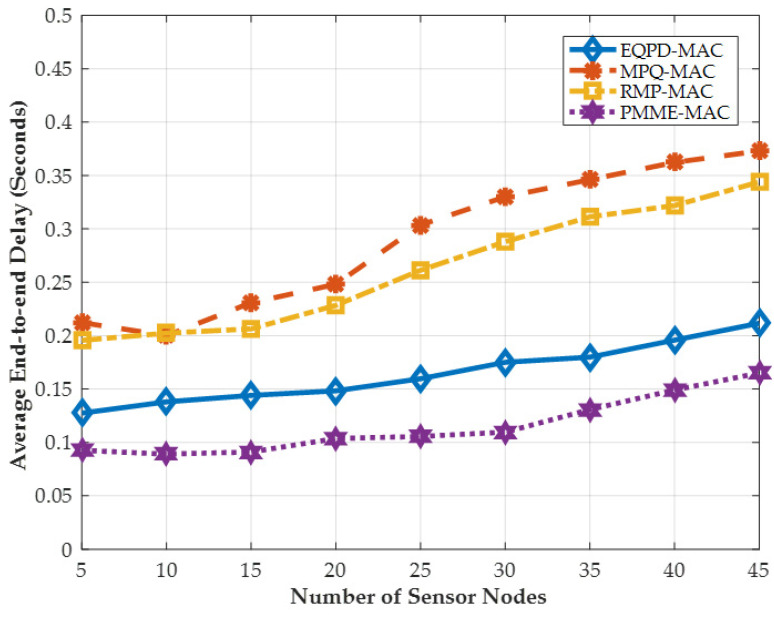
Latency between all packets on an average end-to-end basis.

Figure 12 provides the results of the average end-to-end latency. EQPD-MAC outperforms MPQ-MAC and RMP-MAC, achieving a reduced end-to-end latency of up to 76% and 61%, respectively. It can be seen that MPQ-MAC has the highest latency because the protocol requires nodes to wait longer for the *T_w_* waiting timer to expire, resulting in increased latency. RMP-MAC also has a high latency because as the number of sensor nodes increases, the buffer limit is constantly exceeded, forcing the sensor nodes carrying the data packets to wait longer for the medium to become available. It can also be observed that the PMME-MAC protocol outperforms other protocols in terms of average end-to-end latency. When a sensor node sends a Tx-beacon with any priority, PMME-MAC stops the waiting timer and immediately begins transmitting, which decreases packet latencies but increases energy consumption for other sensor nodes. The sensor nodes in PMME-MAC, on the other hand, are unable to save power by going to sleep automatically when the channel is busy. Data packet latency in EQPD-MAC is around 24% more than in PMME-MAC, despite the fact that this is still within the permissible range. It is also important to note that EQPD-MAC can provide more than 30% improvements in energy consumption, so it does provide the best tradeoff between latency and energy.

### 4.6. Average Network Throughput

**Figure 13:**
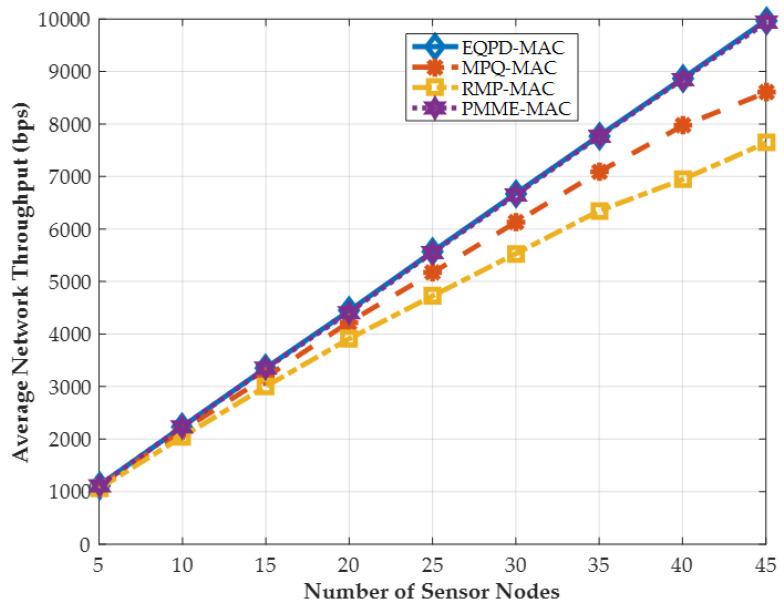
Average network throughput (bps).

Average network throughput, which may be defined as the number of packets received per second, is shown in Figure 13.

(11) $$S=\frac{{NP}_{PktRx}{\times \;L}_{Pkt}}{T_{S}}$$

where *T_S_* denotes the simulation duration in seconds and $L_{Pkt}\;$ is the size of the packet in bytes.

Figure 13 provides the average network throughput. The results show that throughput increases linearly when the number of sensor nodes increases. As can be observed, EQPD-MAC and PMME-MAC provide the maximum throughput among all protocols. EQPD-MAC achieves the highest throughput for any number of sensor nodes deployed in the network, with gains of up to 23.3% and 12%, when compared to RMP-MAC and MPQ-MAC, respectively. Due to EQPD-MAC’s ability to maintain its operation, the sensor nodes can function longer when compared to other protocols. On the other hand, RMP-MAC and MPQ-MAC cannot route a large number of data packets and the network throughput decreases rapidly. Although RMP-MAC protocol achieves good throughput when the number of sensor nodes is small, as the number of sensor nodes rises, the protocol tends to drop packets, resulting in low network throughput.

### 4.7. Packet Delivery Ratio

In the following Equation, the packet delivery ratio (*PDR*) is expressed as follows:

(12) $$PDR=\;\frac{{NP}_{PktRx}}{{NP}_{PktTx}}\;\times \;100\%$$

In this case, the values of ${NP}_{PktRx}$ and ${NP}_{PktTx}$ reflect the total number of data packets received and transmitted, respectively.

**Figure 14:**
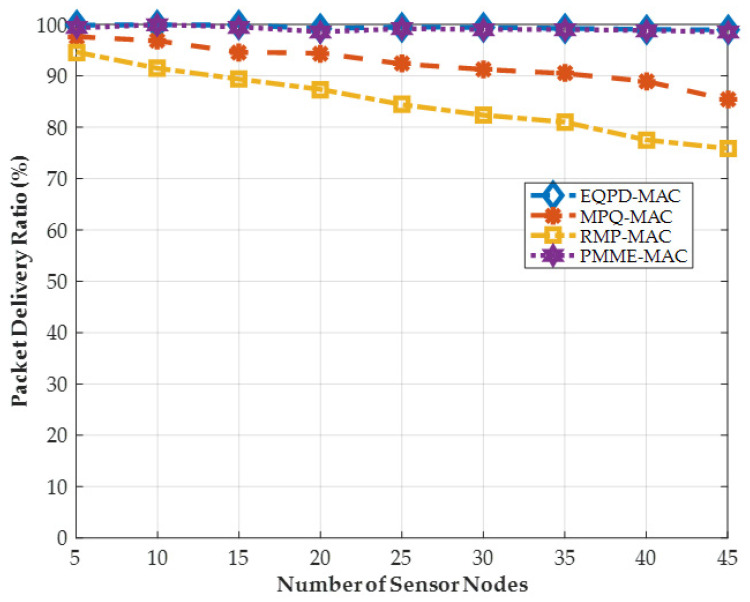
Packet delivery ratio (PDR).

As may be seen in Figure 14, the PDR for each protocol is shown. The results show that EQPD-MAC protocol outperforms RMP-MAC and MPQ-MAC protocols by up to 25% and 14.5%, respectively. In MPQ-MAC and RMP-MAC protocols, the sensor nodes become unresponsive for long periods of time when the buffer limit is exceeded, which results in data packets being dropped. It is also worth noting that in RMP-MAC, the PDR drops considerably as the number of senders increases, indicating that the retransmission limit has been exceeded. The proposed protocol delivers consistently around 100% PDR, like PMME-MAC, for any number of sensor nodes deployed in the network. The first reason is that EQPD-MAC does not encounter any network disruptions, and the sensor nodes are always ready to receive packets from sensor nodes. Secondly, all of the sensor nodes involved in packet routing are synchronized. To prevent packet loss, the sensor nodes check the TA as soon as it receives the Tx-beacon if it has enough time to successfully receive a packet and sensor nodes can extend the TA if required.

## 5. Conclusions

The purpose of this study is to present an energy-aware QoS MAC protocol that maximizes energy efficiency while taking into account multiple levels of data packet priority and multi-hop routing. In the proposed EQPD-MAC protocol, the use of multi-hop routing makes the network deployment more practical and realistic. EQPD-MAC transmits the higher priority packets first, dynamically adjusts listen and sleep time, and incorporates a proven routing technique. A simple and efficient cross-layer communication technique is provided consisting of the Application, Network, and MAC layers. The findings reveal that the EQPD-MAC protocol reduces the power consumption of the sensor nodes while maintaining its network functionality for a longer period than the other investigated protocols. EQPD-MAC protocol outperforms in terms of average energy consumption of the sensor nodes and the sink node, the network throughput, and the PDR. The proposed EQPD-MAC gives the best performance, especially with a high number of sensor nodes. The proposed MAC protocol decreases the energy consumption of sensor nodes by up to 30.3%, the consumption per bit by up to 29.6%, the energy consumption of sink nodes by up to 27.4% and increases the network throughput by up to 23.3%. It also provides an acceptable latency for priority packets while maintaining the lowest energy consumption.

## Figures and Tables

**Table 1:** Priority QoS requirements for various applications.

Applications	Information	Priority QoS Requirements
Monitoring in Smart Cities	Gas Leakage, Health Monitoring, Smoke and Fire	Urgent
	Flood, Security Monitoring	Very Important
	Temperature Monitoring, Traffic Surveillance	Important
	Air Quality Index	Normal
Environmental Monitoring	Volcanic Eruption	Urgent
	Forest Fire	Urgent
	Under Water Monitoring	Important
	Sea Water Level Monitoring	Important
Industry Systems	Safety or Fire Emergency, Hazardous Gas Leakage	Urgent
	Security Monitoring	Very Important
	Equipment Malfunction	Important
	Monitoring	Normal

**Table 2:** Comparative analysis of MAC protocols in WSNs.

Protocol	Multi-Hop	Priority	Energy Efficient
QAEE-MAC [31]	No	Yes	No
MPQ-MAC [30]	No	Yes	No
PMME-MAC [32]	No	Yes	No
RMP-MAC [33]	No	Yes	Yes
EAMP-AIDC [28]	Yes	Yes	No
SPEECH-MAC [36]	Yes	Yes	No
MDA-SMAC [35]	Yes	No	Yes
EDS-MAC [37]	Yes	Yes	No

**Table 3:** Assignment of Priority Packets.

Priority	Data Packet Category
P_1_	Normal
P_2_	Important
P_3_	Very Important
P_4_	Urgent

**Table 4:** Assignment of Priority Packets.

Priority	*R*	*p* (Linear)
P_1_	0.75 < R ≤ 1	0.1
P_2_	0.5 < R ≤ 0.75	0.2
P_3_	0.25 < R ≤ 0.5	0.3
P_4_	0 < R ≤ 0.25	0.4

**Table 5:** Simulation Parameters.

Parameter	Value
Area	200 m × 200 m
Sensor Nodes	45
Radio	CC2420
Simulation Time	10000 s
Frame Time	125 ms
Activation Time (TA)	12 ms
CCA	0.128 ms
Waiting Timeout (*T_w_*)	5 ms
Data Rate	250 kbps
Packet Rate	1 packet/s
SIFS	0.192 ms
Size of Tx-beacon	14 bytes
Size of Rx-beacon	13 bytes
Size of SYNC Packet	5 bytes
Size of Data Packet	28 bytes
Size of ACK Packet	11 bytes
Initial Energy	18738 J
Buffer Size	100 packets
Retransmission limit	10
